# GLUT1 expression patterns in different Hodgkin lymphoma subtypes and progressively transformed germinal centers

**DOI:** 10.1186/1471-2407-12-586

**Published:** 2012-12-10

**Authors:** Sylvia Hartmann, Claudio Agostinelli, Jürgen Diener, Claudia Döring, Stefano Fanti, Pier Luigi Zinzani, Andrea Gallamini, Lothar Bergmann, Stefano Pileri, Martin-Leo Hansmann

**Affiliations:** 1 Senckenberg Institute of Pathology, Goethe University, Frankfurt am Main, Germany; 2 Department of Haematology and Oncological Sciences “L. and A. Seràgnoli”, Haematopathology Section, S. Orsola-Malpighi Hospital, University of Bologna, Bologna, Italy; 3 Department of Nuclear Medicine, Goethe University, Frankfurt am Main, Germany; 4 Department of Nuclear Medicine, Policlinico Sant’Orsola-Malpighi, Bologna University, Via Massarenti 9, Bologna 40138, Italy; 5 Institute of Hematology and Medical Oncology L. e A. Seràgnoli, Policlinico Sant'Orsola-Malpighi, University of Bologna, Bologna, Italy; 6 Hematology Department and BMT Unit, Azienda Ospedaliera S. Croce e Carle, Cuneo, Italy; 7 Department of Haematology, Goethe University, Frankfurt am Main, Germany

**Keywords:** Hodgkin lymphoma, GLUT1, Glycolysis, Warburg effect

## Abstract

**Background:**

Increased glycolytic activity is a hallmark of cancer, allowing staging and restaging with ^18^F-fluorodeoxyglucose-positron-emission-tomography (PET). Since interim-PET is an important prognostic tool in Hodgkin lymphoma (HL), the aim of this study was to investigate the expression of proteins involved in the regulation of glucose metabolism in the different HL subtypes and their impact on clinical outcome.

**Methods:**

Lymph node biopsies from 54 HL cases and reactive lymphoid tissue were stained for glucose transporter 1 (GLUT1), lactate dehydrogenase A (LDHA) and lactate exporter proteins MCT1 and MCT4. In a second series, samples from additional 153 HL cases with available clinical data were stained for GLUT1 and LDHA.

**Results:**

Membrane bound GLUT1 expression was frequently observed in the tumor cells of HL (49% of all cases) but showed a broad variety between the different Hodgkin lymphoma subtypes: Nodular sclerosing HL subtype displayed a membrane bound GLUT1 expression in the Hodgkin-and Reed-Sternberg cells in 56% of the cases. However, membrane bound GLUT1 expression was more rarely observed in tumor cells of lymphocyte rich classical HL subtype (30%) or nodular lymphocyte predominant HL subtype (15%). Interestingly, in both of these lymphocyte rich HL subtypes as well as in progressively transformed germinal centers, reactive B cells displayed strong expression of GLUT1. LDHA, acting downstream of glycolysis, was also expressed in 44% of all cases. We evaluated the prognostic value of different GLUT1 and LDHA expression patterns; however, no significant differences in progression free or overall survival were found between patients exhibiting different GLUT1 or LDHA expression patterns. There was no correlation between GLUT1 expression in HRS cells and PET standard uptake values.

**Conclusions:**

In a large number of cases, HRS cells in classical HL express high levels of GLUT1 and LDHA indicating glycolytic activity in the tumor cells. Although interim-PET is an important prognostic tool, a predictive value of GLUT1 or LDHA staining of the primary diagnostic biopsy could not be demonstrated. However, we observed GLUT1 expression in progressively transformed germinal centers and hyperplastic follicles, explaining false positive results in PET. Therefore, PET findings suggestive of HL relapse should always be confirmed by histology.

## Background

Hodgkin lymphoma (HL) is a common lymphoma in the Western world. Most individuals affected are adolescents. The tumor cells in HL, the Hodgkin- and Reed-Sternberg (HRS) cells, are derived from germinal center B cells [1], which have lost their B cell phenotype [2]. HRS cells represent only a minority in their reactive microenvironment, which is mainly composed of T cells, epithelioid histiocytes, and eosinophils [3]. Several signaling pathways have been found to be constitutively deregulated in HL including NF-kappaB, JAK-STAT, PI3K-Akt, ERK, AP1, NOTCH1 and receptor tyrosine kinases [4-8].

A hallmark of malignant tumors is the “Warburg effect”—high glucose consumption and increased glycolytic activity [9,10], which occurs as cancer cells shift their metabolism from oxidative phosphorylation to much less efficient glycolysis independent of their oxygen supply [11]. This needs to be compensated by increased glucoseconsumption. High glucose uptake by malignant tumors is the pathophysiologic basis for imaging with 18F-fluorodeoxyglucose (FDG) positron emission tomography (PET). Thus, different PET studies have shown high FDG uptake in HL [12,13]. Interim-PET, accomplished after the first cycles of chemotherapy, has proven to be an important prognostic tool in HL [14-16]. Energy independent glucose uptake into malignant and non-malignant cells is regulated via the expression of glucose transporter (GLUT) proteins [17,18]. GLUT1 and GLUT3, which are both members of the *SLC2A* group, have high affinity for glucose [19]. HRS cells were shown to express GLUT1, whereas GLUT3 and Hexokinase II were either not expressed or were only weakly expressed [20,21]. Because a correlation between GLUT1 protein expression in lymphoma cells and FDG uptake in PET scan was recently demonstrated [20-23], we investigated the possibility that GLUT1 expression in HRS cells might also predict clinical behavior.

In the cytoplasm, glucose is cleaved by glycolytic enzymes into two molecules of pyruvate and is then transformed into lactate by lactate dehydrogenase (LDH), which is a tetramer composed of two different polypeptide chains, LDHA and LDHB. Whereas LDHA favors the conversion of pyruvate into lactate, LDHB is more efficient in converting lactate into pyruvate [24]. Lactate efflux results via membrane-bound monocarboxylate transporters (MCT) such as MCT1-4. MCT1 and 4 have low affinity for lactate, which makes them ideal export molecules when the metabolite is generated at high levels in the cytoplasm [24].

In the current study, we investigated the expression of GLUT1, LDHA, MCT1 and MCT4 in tumor cells and reactive bystander cells in different HL subtypes and in respect to treatment outcome in a large number of patients. All four proteins would be expected to be highly expressed in cells exhibiting a high level of glucose metabolism, but with exception of GLUT1, to our knowledge, have not been investigated in Hodgkin lymphoma.

## Methods

Tissues investigated consisted of paraffin embedded whole tissue sections of normal lymphoid tissue (tonsils) and 54 HL samples obtained from the archive files of the Senckenberg Institute, which were stained for CD20, CD3, CD15, LMP1, GLUT1, LDHA, MCT1 and MCT4 (patient details in Table 1). Routine pre-chemotherapy PET data were available for eight patients. Furthermore, 10 lymph nodes with progressively transformed germinal centers (PTGC) were stained for GLUT1. An additional series of 153 classical HL with available clinical and PET data were obtained from the Hematopathology Section, S. Orsola-Malpighi Hospital, University of Bologna, and stained in triplicate on tissue microarray (TMA) format for GLUT1 and LDHA (patient details in Table 1). All patients underwent pre-chemotherapy PET and Interim PET after the two initial cycles of ABVD. SUVmax values at the biopsy site of pre-chemotherapy PET data were available in 13 patients. All patients were treated with Doxorubicin, Bleomycin, Vinblastine and Dacarbazine (ABVD). Patients underwent 2 or 6 courses of ABVD depending on the disease stage. No treatment change was made based on interim PET scan results. For the pre-chemotherapy PET, a quantitative assessment of FDG uptake was made by calculating the maximum standard uptake value (SUVmax) in a region of interest within the nodal or extranodal site showing the highest intensity of FDG uptake. Immunostainings were performed as previously described [25] and were simultaneously assessed by two pathologists (S.H., M.L.H) on a multi-head microscope. The antibodies and detailed methods are listed in Additional file 1: Table S1. Immunohistochemical stainings and PET scans were reviewed without knowledge of other findings.

**Table 1:** Clinical and pathological characteristics of the Hodgkin lymphoma patients studied for GLUT1 and LDHA expression

	**HL cases**	**Membrane bound GLUT1 expression**	**Cytoplasmic LDHA expression**
N	207	102/207 (49%)	68/153 (44%)
Age, median in years	34		
Gender, male (%)	48		
Histology			
-Nodular sclerosis	140	79/140 (56%)	44/97 (45%)
-Mixed cellularity	29	12/29 (41%)	8/22 (36%)
-Lymphocyte-rich	10	3/10 (30%)	3/10 (30%)
-Lymphocyte-depleted	6	6/6 (100%)	4/6 (67%)
-NOS (not classifiable)	9	0/9 (0%)	3/5 (60%)
-Lymphocyte predominant	13	2/13 (15%)	6/13 (46%)
Stage (%)			
- I	2		
- II	89		
- III	37		
- IV	25		
B-Symptoms (%)	46		
Bulky disease (%)	32		
Interim-PET positive (%)	18		
Treatment			
- ABVD (%)	80		
- ABVD + radiation (%)	20		

Informed consent was obtained in accordance with the Declaration of Helsinki and approval was obtained from the ethics committees of the University Hospitals of Frankfurt and Bologna. Statistical analyses were performed using the statistical computing environment R [26]. The data displayed a Gaussian distribution and for pairwise comparisons Fisher's Exact Test was used. Progression free survival (PFS) and overall survival (OS) were estimated according to Kaplan-Meier [27]. PFS was defined as the time from diagnosis to disease progression or death from any cause. OS was defined as the time from diagnosis to death of any cause.

## Results and discussion

### Staining patterns in reactive tissue

**Figure 1:**
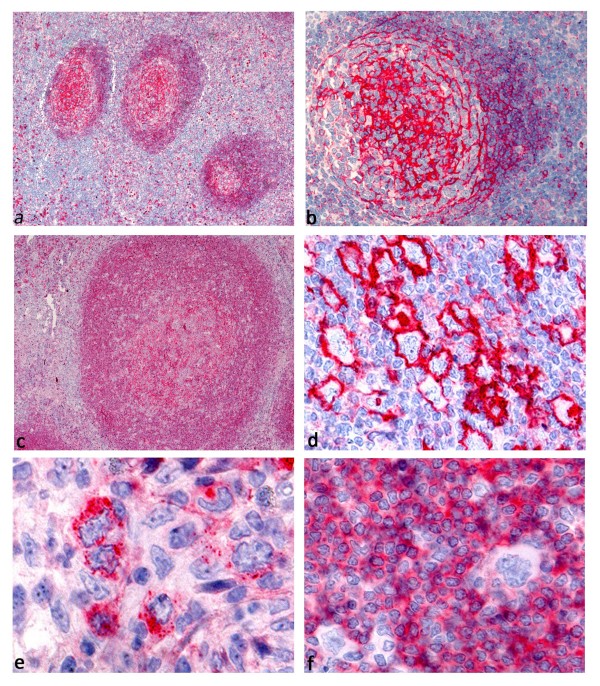
**GLUT1 immunostaining.a**, **b**. Reactive germinal centers showing membrane bound GLUT1 expression in follicular dendritic cells. **c**. GLUT1 expression in the enlarged mantle zone of a progressively transformed germinal center. **d**. NSCHL with membrane bound GLUT1 expression by the HRS cells. **e**. NSCHL with granular cytoplasmic expression of GLUT1 by the HRS cells. **f**. NLPHL with GLUT1 expression in the reactive B cells, but no GLUT1 expression in the LP cells.

In reactive tonsils, follicular dendritic cells (FDCs) in the germinal centers as well as interfollicular plasma cells exhibited strong membrane bound expression of GLUT1, whereas small B lymphocytes in the mantle zone showed moderate GLUT1 expression in all cases (Figure 1a,b). Additionally, few interfollicular blasts, as well as basal layers of squamous epithelium showed membrane bound GLUT1 expression. No GLUT1 expression was observed in CD3-positive lymphocytes in T cell areas. B cells in the enlarged mantle zone in PTGC showed moderate GLUT1 expression, which may explain false positive results in PET imaging [28,29]. LDHA was expressed in most germinal center blasts, whereas interfollicular areas remained negative. Membrane bound MCT1 expression was found in very few germinal center blasts and basal squamous epithelial layers. Interfollicular areas did not stain for MCT1. Following a pattern similar to GLUT1, MCT4 was expressed by FDCs in the germinal centers, but was not expressed in the mantle zones. Additionally, basal squamous epithelial layers exhibited membrane bound MCT4 expression.

### GLUT1 expression in HL

Membrane bound GLUT1 expression was observed on HRS cells in 102 of 207 HL cases (49%, Figure 1, Table 1). Whereas membrane bound GLUT1 expression was observed more frequently in lymphocyte depleted HL (LDCHL,100%), nodular sclerosing HL (NSCHL, 56%) and mixed cellularity HL (MCCHL, 41%), membrane bound GLUT1 expression was observed in only 15% of nodular lymphocyte predominant HL (NLPHL) and in 30% of lymphocyte rich classical HL (LRCHL). This is in line with the study by Khandani et al. [20] in which no GLUT1 expression was found in the LP cells in two NLPHL investigated, whereas GLUT1 expression was observed in three NSCHL. Shim et al. [21] found expression of GLUT1 in 10 to 90% of HRS cells in four classical HL, including one LDCHL, one NSCHL and two MCCHL; the highest rate was observed in the NSCHL and the lowest rate in the LDCHL. Glucose uptake and metabolism in the tumor cells may, therefore, be less important in LP cells of NLPHL than in HRS cells of classical HL, as suggested by Hutchings et al., who, in a PET study, found significantly lower standardized glucose uptake values in NLPHL than in classical HL [13]. Nonetheless, a different study showed that staging by PET scan is applicable in NLPHL [30]. We observed GLUT1 expression in the reactive B cell infiltrate in lymphocyte rich HL subtypes (Figure 1f), while the T cell dominated areas were GLUT1-negative. If membrane bound GLUT1 expression was observed in the HRS cells, then GLUT1 was present in almost 100% of the tumor cells regardless of the localization of the HRS cells in the tissue. This indicates that HRS cells themselves possibly contribute to the PET positivity of HL in these cases via their GLUT1 expression, although the tumor cells may be a minority in the infiltrate. On the other hand, PET positivity of NLPHL is likely due to the strong glucose uptake in the reactive B cells.

We then asked if different patterns of GLUT1 expression (negative, cytoplasmic or membrane bound) in the primary biopsy had predictive value regarding progression free survival and overall survival. However, we found no significant prognostic value of the different GLUT1 staining patterns (Additional file 2: Figure S1), in contrast to the powerful prognostic tool of interim-PET [16] and in contrast to the prognostic impact of GLUT1 expression found in other cancer types [31,32]. We also evaluated the possibility that different patterns of GLUT1 expression could predict the interim-PET result. However, no significant association between the number of interim-PET positive cases and the pattern of GLUT1 staining was found (Fisher's Exact Test, p = 0.6).

SUVmax values of pre-chemotherapy PET scans were available for eight patients of the first series (stained on full sections) and for 13 patients of the second series (stained on TMA format). SUVmax values in the first series were low to moderate (range 2.55 – 12.8), and a tendency, albeit not significant, toward higher SUVmax values in cases with membrane bound GLUT1 expression was observed in the first series (Additional file 3: Figure S2). In the second series the SUVmax values were generally higher (range 4.1 – 18.9), but in this series, no correlation between GLUT1 staining results and SUVmax values was observed. This may be simply a consequence of the low number of cases with available pre-chemotherapy PET data in either series. As well, immunohistochemical staining on TMA may not be comparable to staining of full sections. There may be different mechanisms of glucose uptake, not only in the tumor cells, but also in the reactive bystander cells. Potential alternative transporters like GLUT3 and GLUT4 [33] were not investigated in the present study. However, Khandani et al. [20] observed GLUT3 expression in approximately 20% of the cells in the microenvironment. It is possible, therefore, that different mechanisms of glucose uptake, e.g., via GLUT1 in HRS cells and via GLUT3 in the microenvironment, contribute to PET positivity of the tumor.

### Expression of LDHA and lactate transporter proteins in HL

**Figure 2:**
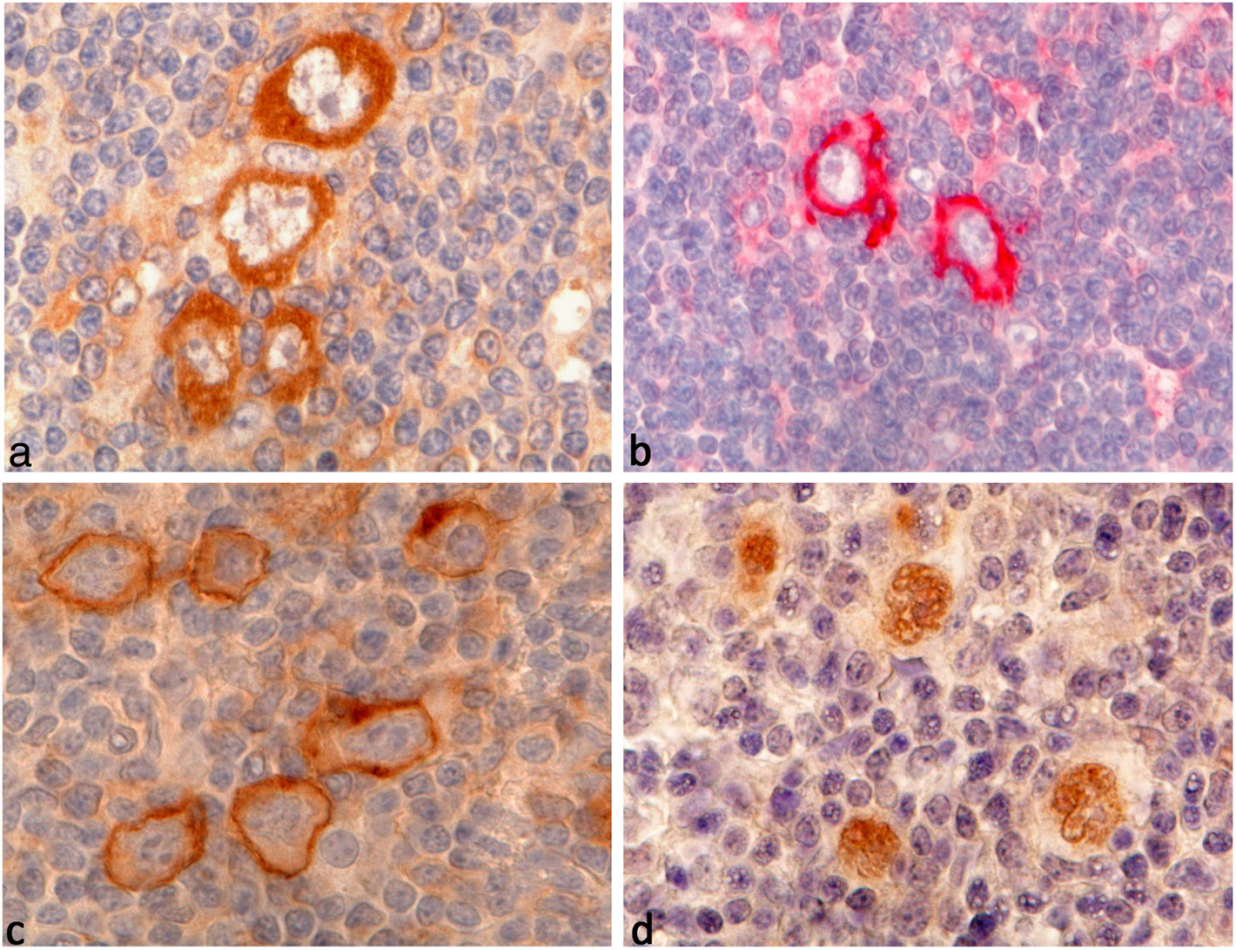
**LDHA**, **MCT4 and MCT1 immunostainings.a**. NSCHL with strong cytoplasmic LDHA staining in the HRS cells. **b**. NLPHL with membrane bound expression of MCT4 in the LP cells. **c**. NSCHL with membrane bound expression of MCT1 in the HRS cells. **d**. LRCHL with nuclear staining for MCT1 in the HRS cells.

Since membrane bound expression of GLUT1 suggests increased glycolysis, the expression levels of LDHA, an enzyme acting downstream of glycolysis, and of two lactate transporters were investigated in the same HL cases. Cytoplasmic LDHA expression was observed in a 68 out of 153 HL cases (44%, Table 1, Figure 2), with the highest number of positive cases in the LDCHL subtype (67%). However, no significant differences were observed with respect to progression free survival and overall survival (Additional file 2: Figure S1). This was somewhat surprising because high serum LDH levels are known to be an adverse prognostic factor for Hodgkin lymphoma patients [34].

As lactate must finally be exported from the cell, the expression of two transporter proteins for lactate and pyruvate, MCT1 and MCT4, were investigated in the first series of cases. Compared to the number of cases expressing GLUT1 and/or LDHA, only a few cases exhibited membrane bound MCT1 or MCT4 expression (Figure 2). MCT4 was expressed by tumor cells in 10 of 54 HL cases (19%). Cases that did not show MCT4 expression by tumor cells exhibited MCT4 expression in macrophages and epithelioid cells. Membrane bound expression of MCT1 was observed in 13 of 54 HL cases (24%). Cytoplasmic as well as nuclear MCT1 expression occurred in 13 (24%) and 4 cases (7%), respectively (Figure 2). There have been conflicting data regarding the expression of MCT1 and MCT4 in cancer. These were expressed in only few cases in the present study. Similar to our observations, an inverse correlation of GLUT1 and MCT1 expression was described in colonic cancer [35]. Thus, lactate efflux appears to only play a minor role in classical HL, possibly because the dumping of excess carbon as lactate allows more rapid incorporation of carbon into biomass.

## Conclusions

We observed GLUT1 and/or LDHA expression in the HRS cells of a large number of classical HL cases indicating high glycolytic activity of the tumor cells. Nevertheless, the expression pattern of these proteins did not predict prognosis or survival, nor did it correlate with interim-PET results. Powerful prognostic tools like interim-PET cannot, therefore, be replaced by applying these immunohistochemical stainings. However, we also observed GLUT1 expression in progressively transformed germinal centers and hyperplastic follicles, which can explain false positive results in PET. Therefore, PET findings suggestive of HL relapse should always be confirmed by histology.

## Competing interests

The authors report no potential conflict of interest.

## Authors' contributions

SH: Immunostainings, histological evaluation, analysis and interpretation of data, drafting the manuscript; CA: acquisition of patients and clinical data, pathologic review, TMA construction, JD, SF: performance and interpretation of PET scans; CD: statistical analysis of the data, LB, PLZ, AG: acquisition and interpretation of clinical data of patients; SAP: acquisition of patients and clinical data, pathologic review, MLH: histological evaluation, experimental design, revising the manuscript. All authors read and approved the final manuscript.

## Pre-publication history

The pre-publication history for this paper can be accessed here:

http://www.biomedcentral.com/1471-2407/12/586/prepub

## Supplementary Material

Additional file 1 **Table S1.** Antibodies, dilutions, suppliers and detection systems used in the present study. Click here for file

Additional file 2 **Figure S1.** Kaplan-meier Analysis of GLUT1 and LDHA expression as well as Interim PET. a. Kaplan-Meier Analysis of different GLUT1 expression patterns (overall survival). b. Kaplan-Meier Analysis of different GLUT1 expression patterns (progression free survival). c. Kaplan-Meier Analysis of LDHA expression (overall survival). d. Kaplan-Meier Analysis of LDHA expression (progression free survival). e. Kaplan-Meier-Analysis of Interim-PET-positive and -negative cases (overall survival). f. Kaplan-Meier-Analysis of Interim-PET-positive and -negative cases (progression free survival). Click here for file

Additional file 3 **Figure S2.** SUVmax in different GLUT1 expression patterns. a. Prechemotheraphy SUVmax values in 8 patients of the first series stained on full sections. b. Prechemotheraphy SUVmax values in 13 patients of the second series stained on TMA format. Click here for file
