# Supplementary material for: GLUT1 expression patterns in different Hodgkin lymphoma subtypes and progressively transformed germinal centers
Source: BMC Cancer. 2012 Dec 10;12:586. doi: 10.1186/1471-2407-12-586 (PMC3537691; doi:10.1186/1471-2407-12-586)
Supplement: Additional file 1 — Table S1. Antibodies, dilutions, suppliers and detection systems used in the present study. [file 1471-2407-12-586-S1.doc]

| Antibody | Dilution | Provider | Detection System used |
| --- | --- | --- | --- |
| CD3 | 1:200 | Novocastra, Newcastle upon Tyne, UK | Dako REAL Detection System (Alkaline Phosphatase/RED, K5005, DAKO, Glostrup, Denmark) |
| CD20 | 1:1000 | DAKO | Dako REAL Detection System |
| GLUT1/SPM498 | 1:200 | Lab Vision, USA | Dako REAL Detection System |
| LDHA (LS-C49934) | 1:100 | Lifespan, Goettingen, Germany | Peroxidase-EnVision Plus (DAKO) |
| MCT1 (HPA003324) | 1:200 | Atlas Antibodies, Stockholm, Sweden  ([www.proteinatlas.org](http://www.proteinatlas.org/)) | Peroxidase-EnVision Plus (DAKO) |
| MCT4 (HPA021451) | 1:25 | Atlas Antibodies  ([www.proteinatlas.org](http://www.proteinatlas.org/)) | Dako REAL Detection System |
| CD15 | 1:1000 | DAKO | Dako REAL Detection System |
| LMP1 (M0897) | 1:200 | DAKO | Peroxidase-EnVision Plus (DAKO) |

**Suppl. Table 1** Antibodies, dilutions, suppliers and detection systems used in the present study.
