# Supplementary figures and images for: GLUT1 expression patterns in different Hodgkin lymphoma subtypes and progressively transformed germinal centers
Source: BMC Cancer. 2012 Dec 10;12:586. doi: 10.1186/1471-2407-12-586 (PMC3537691; doi:10.1186/1471-2407-12-586)

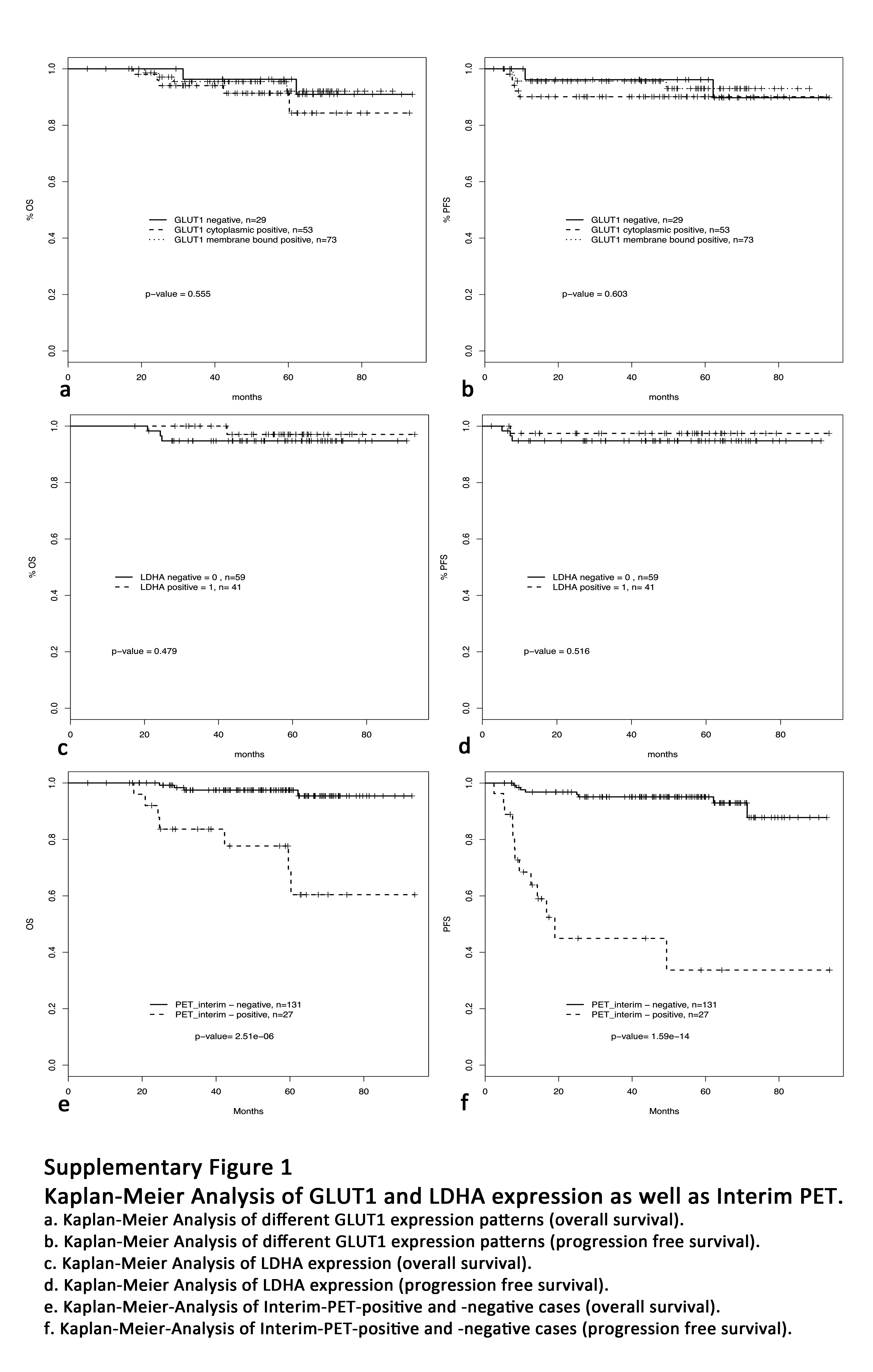

Supplement: Additional file 2 — Figure S1. Kaplan-meier Analysis of GLUT1 and LDHA expression as well as Interim PET. a. Kaplan-Meier Analysis of different GLUT1 expression patterns (overall survival). b. Kaplan-Meier Analysis of different GLUT1 expression patterns (progression free survival). c. Kaplan-Meier Analysis of LDHA expression (overall survival). d. Kaplan-Meier Analysis of LDHA expression (progression free survival). e. Kaplan-Meier-Analysis of Interim-PET-positive and -negative cases (overall survival). f. Kaplan-Meier-Analysis of Interim-PET-positive and -negative cases (progression free survival). [file 1471-2407-12-586-S2.jpeg]

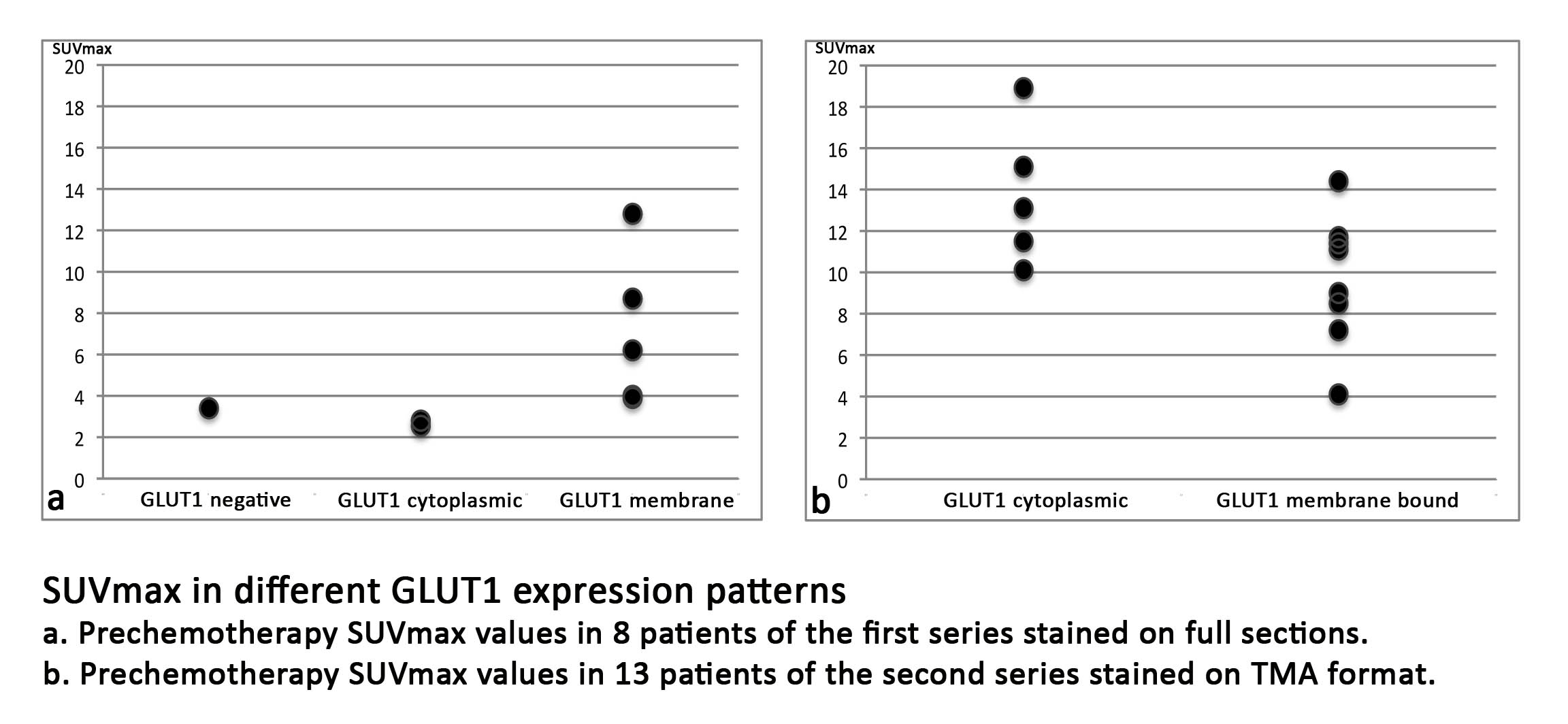

Supplement: Additional file 3 — Figure S2. SUVmax in different GLUT1 expression patterns. a. Prechemotheraphy SUVmax values in 8 patients of the first series stained on full sections. b. Prechemotheraphy SUVmax values in 13 patients of the second series stained on TMA format. [file 1471-2407-12-586-S3.jpeg]
